# Determinants of Vaccination Coverage and Consequences for Rabies Control in Bali, Indonesia

**DOI:** 10.3389/fvets.2016.00123

**Published:** 2017-01-09

**Authors:** Riana A. Arief, Katie Hampson, Andri Jatikusumah, Maria D. W. Widyastuti, Sunandar, Chaerul Basri, Anak A. G. Putra, Iwan Willyanto, Agnes T. S. Estoepangestie, I. W. Mardiana, I. K. G. N. Kesuma, I. P. Sumantra, Paul F. Doherty, M. D. Salman, Jeff Gilbert, Fred Unger

**Affiliations:** ^1^Center for Indonesian Veterinary Analytical Studies, Bogor, Indonesia; ^2^Boyd Orr Centre for Population and Ecosystem Health, Institute of Biodiversity, Animal Health and Comparative Medicine, University of Glasgow, Glasgow, UK; ^3^Department of Animal Disease and Veterinary Public Health, Faculty of Veterinary Medicine, Bogor Agricultural University, Bogor, Indonesia; ^4^Denpasar Disease Investigation Center, Denpasar, Indonesia; ^5^InI Veterinary Service, Surabaya, Indonesia; ^6^Department of Veterinary Public Health, Faculty of Veterinary Medicine, Airlangga University, Surabaya, Indonesia; ^7^Bali Provincial Livestock and Animal Health Office, Denpasar, Indonesia; ^8^Department of Fish, Wildlife and Conservation Biology, Warner College of Natural Resources, Colorado State University, Fort Collins, CO, USA; ^9^Animal Population Health Institute, College of Veterinary Medicine and Biomedical Sciences, Colorado State University, Fort Collins, CO, USA; ^10^International Livestock Research Institute, Hanoi, Vietnam

**Keywords:** rabies, dogs, vaccination, questionnaire survey, mark–recapture survey, Bali, Indonesia

## Abstract

Maintaining high vaccination coverage is key to successful rabies control, but mass dog vaccination can be challenging and population turnover erodes coverage. Declines in rabies incidence following successive island-wide vaccination campaigns in Bali suggest that prospects for controlling and ultimately eliminating rabies are good. Rabies, however, has continued to circulate at low levels. In the push to eliminate rabies from Bali, high coverage needs to be maintained across all areas of the island. We carried out door-to-door (DTD) questionnaire surveys (*n* = 10,352 dog-owning households) and photographic mark–recapture surveys (536 line transects, 2,597 observations of free-roaming dogs) in 2011–2012 to estimate dog population sizes and assess rabies vaccination coverage and dog demographic characteristics in Bali, Indonesia. The median number of dogs per subvillage unit (*banjar*) was 43 (range 0–307) for owned dogs estimated from the DTD survey and 17 (range 0–83) for unconfined dogs (including both owned and unowned) from transects. Vaccination coverage of owned dogs was significantly higher in adults (91.4%) compared to juveniles (<1 year, 43.9%), likely due to insufficient targeting of pups and from puppies born subsequent to vaccination campaigns. Juveniles had a 10–70 times greater risk of not being vaccinated in urban, suburban, and rural areas [combined odds ratios (ORs): 9.9–71.1, 95% CI: 8.6–96.0]. Free-roaming owned dogs were also 2–3 times more likely to be not vaccinated compared to those confined (combined Ors: 1.9–3.6, 95% CI: 1.4–5.4), with more dogs being confined in urban (71.2%) than in suburban (16.1%) and rural areas (8.0%). Vaccination coverage estimates from transects were also much lower (30.9%) than household surveys (83.6%), possibly due to loss of collars used to identify the vaccination status of free-roaming dogs, but these unconfined dogs may also include dogs that were unowned or more difficult to vaccinate. Overall, coverage levels were high in the owned dog population, but for future campaigns in Bali to have the highest chance of eliminating rabies, concerted effort should be made to vaccinate free-roaming dogs particularly in suburban and rural areas, with advertising to ensure that owners vaccinate pups. Long-lasting, cheap, and quick methods are needed to mark vaccinated animals and reassure communities of the reach of vaccination campaigns.

## Introduction

Dog vaccination is a valuable component for building high herd immunity with the aim to prevent the spread of rabies and when deployed effectively can eliminate infection (1, 2). The most critical factors that determine the effectiveness of vaccination are the level of vaccination coverage achieved and the comprehensiveness of campaigns (3–5).

Campaigns that vaccinate at least 70% of the dog population are considered necessary to eliminate infection (6). However, population turnover poses a challenge to maintaining sufficient levels of vaccination coverage (7). Declines in vaccination coverage occur as vaccinated animals die and susceptible puppies are born, or new unvaccinated animals are brought into the population. Therefore, in the aftermath of a vaccination campaign, coverage can rapidly decline. Furthermore, even relatively small gaps in vaccination coverage can facilitate the persistence of infection (4, 5). Understanding the obstacles to maintain high vaccination coverage between campaigns is therefore critical to ensure the elimination of rabies from Bali.

The island of Bali, Indonesia, was historically rabies free. In 2008, however, an incursion occurred, and the disease spread rapidly across the island. The resulting epidemic led to over 100 human rabies deaths (8) and thousands of human exposures requiring expensive postexposure prophylaxis (9). Local, national, and international pressure led to concerted efforts to control the disease, largely based on mass vaccination of dogs, but also including culling of dogs by local authorities, as described by Putra et al. (9). The first dog vaccination campaign was conducted from October 2010 to March 2011, followed by the second campaign in April through June 2011, and third campaign in March to June 2012 (10, 11). Over 249,000 dogs were vaccinated in the first campaign with an estimated coverage of 77%, while in the second campaign, over 244,000 dogs were vaccinated with 74% coverage (10, 12). A marked decrease in rabies cases in dogs and human was observed after each campaign (12). Subsequent campaigns were conducted annually starting in April and ending in June or July of the same year.

Here, we assess dog population characteristics affecting the level of vaccination coverage achieved in different settings and segments of the dog population in Bali. We discuss how these differences in coverage arise and what challenges they pose to improve vaccination delivery. Our findings have immediate implications for improving rabies control efforts on Bali, and wider application to other densely populated areas where rabies circulates in large populations of mainly free-roaming dogs.

## Materials and Methods

### Study Area

**Figure 1:**
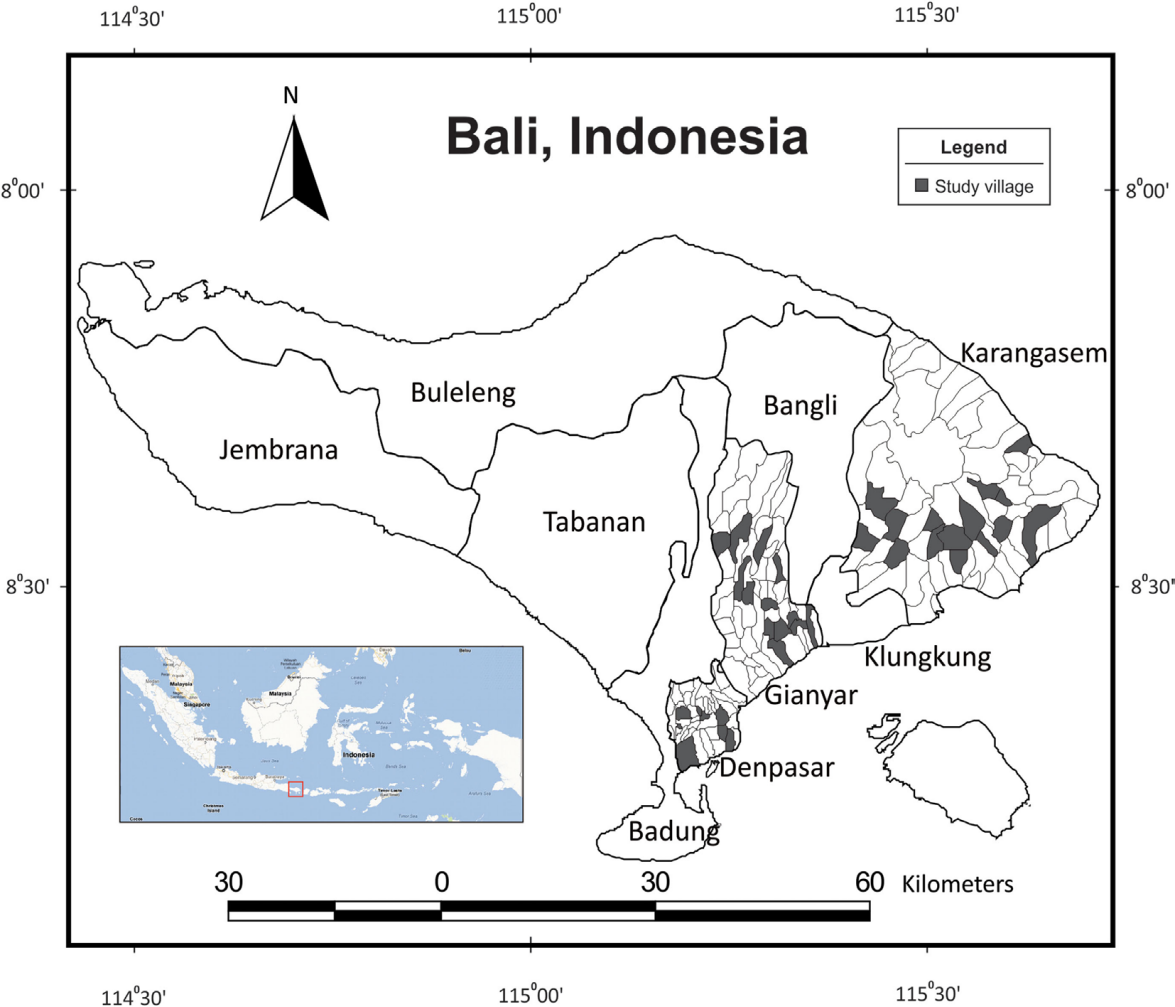
**Surveyed villages (*n* = 37) in Denpasar, Gianyar, and Karangasem regencies on the island of Bali, Indonesia (see inset)**.

The research was carried out between March 2011 and February 2012 in three of the nine regencies in Bali, Indonesia (Figure 1). Denpasar, Gianyar, and Karangasem regencies were generally selected to represent urban, suburban, and rural areas, respectively. Surveys were conducted after the first rabies vaccination campaign (October 2010–March 2011) and through the second campaign (April–June 2011) on the island due to the large number of villages involved (10, 11). All surveys were carried out within a year after either vaccination campaign took place.

Two approaches were used to estimate demographic parameters and levels of vaccination coverage for owned dogs and free-roaming dogs: door-to-door (DTD) household surveys and line transects with photographic mark–recapture (PMR) methods. Data on owned dogs were collected using the DTD survey, while data on unconfined or free-roaming dogs that included both owned or stray/feral dogs were collected using the PMR transects. The approaches were selected to obtain information from the entire dog population in Bali despite the likelihood of overlap between owned dogs and unconfined free-roaming dog populations (13, 14).

**Figure 2:**
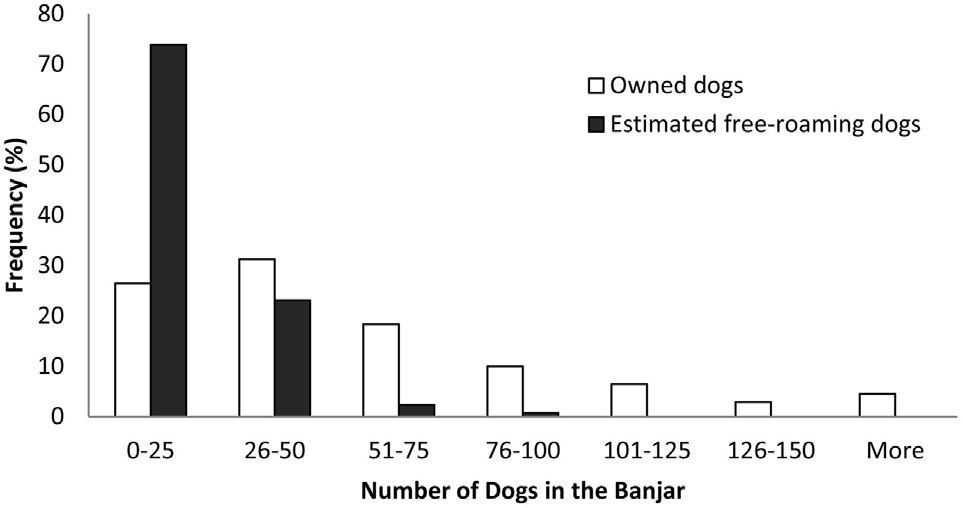
**Distribution of the number of owned dogs (*n* = 310 *banjars*) and estimated free-roaming dogs (*n* = 130 *banjars*) in *banjars***.

The sampling unit for both methods was the *banjar*, a local cultural and government unit. One village consists of as few as 2 and as many as 17 *banjars*. A two-stage sampling design was used to sample the *banjars*. First, 37 villages were randomly selected, proportional to the number of villages in each regency. Subsequently, all *banjars* in selected villages were included in the DTD survey, and four *banjars* per village were randomly selected for the PMR survey. One selected village only had two *banjars*, thus all *banjars* were included in both surveys. Information on main occupations and public facilities in villages were used to post-stratify *banjars* into urban, suburban, and rural categories. In urban villages, majority of the occupations are non-agricultural and public facilities, such as markets, public transport, schools, and government offices, are more prevalent; the opposite is found in rural villages (15). Descriptive information on *banjar* was 43 (range 0–307) (Figure 2) features considered likely to affect dog populations, including the presence of markets, bus terminals, temples, schools, beaches, rice paddies, plantations, or forest, were collected in addition to data on recent dog culling activities within 3 months prior to the survey.

### DTD Survey

We conducted a census of owned dogs by interviewing a member of every dog-owning household in selected *banjars* was 17 (range 0–83) (Figure 2). The interview process was assisted by *banjar* officials (*klian*), who have very strong social relationships with people in the community, resulting in full compliance from all households interviewed. For every reported owned dog, we collected data on the animal’s sex, age group (juveniles <1 year old and adults ≥1 year old), rabies vaccination status, and confinement status as reported by their owner. A dog was defined as confined if its movement and access to public areas were restricted by its owners.

### PMR Survey

In each selected *banjar*, we carried out a total of four daily transects and photographed every free-roaming dog observed within a 25 m radius. A free-roaming dog was identified as a dog whose movement was not restricted by any direct man-made intervention, such as a leash or closed-off fence. Transects were conducted when dogs were most active, alternating between starting in the morning (6:30 a.m.) and the afternoon (4:00 p.m.). Transects covered all main roads and paths in the *banjar*, and routes were planned to avoid any overlap. Motorcycles were used to drive transects at a maximum speed of 10 km/h. Observers stopped every time a dog was sighted to collect at least three photographs. From the photographs, we derived data on dog encounter (first sight or resight), sex, estimated age (juveniles <1 year or adults ≥1 year), and vaccination status (presence/absence of a vaccination collar). Transects were typically completed within 2–3 h.

### Data Analysis

Demographic characteristics of dogs and vaccination coverage of dogs identified through the two survey methods were classified by urban, suburban, and rural strata. Dog was the sampling unit for this analysis. Chi-square and Fisher’s exact tests were used to compare the differences in the proportion of dog demographic characteristics between groups.

Photographic mark–recapture data of free-roaming dogs were analyzed using Huggins closed capture model to derive estimates of population abundance (16, 17). The model allows estimation of the detection probabilities of dogs and subsequent correction of abundance estimates. Independent variables investigated with the model were time, dog sex, dog age, urbanization strata, descriptive *banjar* information, and recent culling activities. Model selection was conducted using Akaike’s information criteria (AIC) with small sample size correction (AICc) and multi-model inference (18). For each variable, AICc cumulative weights were computed. Variables with weights of 0.5 or greater were selected for the final predictive model (19). PMR analysis was conducted using the program MARK version 7.1 (20).

Multiple logistic regression models for the odds of dogs not being vaccinated were built separately for owned dogs and observed free-roaming dogs with urbanization strata, demographic characteristics, descriptive *banjar* information, and recent dog culling activities in *banjar* as independent variables. Purposive selection method derived by Hosmer et al. and AIC were used to establish the final logistic regression model (21). Data manipulation and modeling were conducted using R and R package “multcomp” (22, 23).

## Results

### Dog Demography

During the DTD survey, we visited 310 *banjars*. In all households we visited, household members agreed to participate in the study. We therefore interviewed members of 10,352 dog-owning households and collected data on 17,376 owned dogs. On average, urban *banjars* had 1,318 (SE 70) residents, while suburban and rural *banjars* had fewer residents with 674 (SE 46) and 704 (SE 31) people, respectively. The average number of dogs per dog-owning household was 1.68 (95% CI: 1.66–1.69), and the median number of owned dogs per *banjar* was 43 (range 0–307). In seven *banjars*, none of the households owned any dogs.

In the PMR survey, we completed 536 transects in 130 *banjars* and made 2,597 observations of free-roaming dogs. From these observations, 1,972 individuals were identified. No free-roaming dog was observed in eight *banjars*. The estimated average detection probability was 0.19 and process standard error was 0.024. The median number of estimated free-roaming dogs per *banjar* was 17 (range 0–83.3).

The overall male to female sex ratio was 2.4:1 and 3.3:1 for owned dogs and observed free-roaming dogs, respectively. This male-biased demography was consistent across urban, suburban, and rural populations (Table 1), although not as strong in owned and observed free-roaming dogs in urban areas (*p* < 0.001). Male bias was stronger in owned adult dogs (>1 year, 2.6:1) compared to juveniles (1.6:1) in all areas (urban *p* < 0.001; suburban *p* < 0.001; rural *p* < 0.001). No significant difference in sex ratio was observed between free-roaming adult and juvenile dogs; however, only a small number of free-roaming juveniles were observed (*n* = 68 dogs). The overall sex ratio in free-roaming dogs was 3.3:1 in adults and 4.4:1 in juveniles.

**Table 1:** **Demographic characteristics of owned dogs and observed free-roaming dogs in 37 villages in Bali**.

	Urban	Suburban	Rural	Overall
**Number of dogs**				
Owned dogs	6,605	3,501	7,270	17,376
Observed free-roaming dogs	840	313	819	1,972
**Sex ratio (male:female, 95% CI)**				
Owned dogs	1.7 (1.6, 1.8)	2.6 (2.4, 2.8)	3.2 (3.0, 3.4)	2.4 (2.3, 2.5)
Juvenile (*n* = 2,874)	1.2 (1.1, 1.4)	1.7 (1.4, 2.1)	2.1 (1.9, 2.4)	1.6 (1.5, 1.8)
Adult (*n* = 14,502)	1.8 (1.7, 1.9)	2.8 (2.6, 3.0)	3.5 (3.3, 3.8)	2.6 (2.5, 2.7)
Observed free-roaming dogs^a^	2.4 (2.1, 2.8)	4.2 (3.2, 5.7)	4.6 (3.9, 5.6)	3.3 (3.0, 3.7)
Juvenile (*n* = 65)^b^	–	–	–	4.4 (2.6, 10.2)
Adult (*n* = 1,904)	2.3 (2.0, 2.7)	4.1 (3.2, 5.7)	4.6 (3.9, 5.6)	3.3 (3.0, 3.7)
**Adult, ≥1 year old (%, 95% CI)**				
Owned dogs	81.7 (80.8, 82.6)	87.5 (86.4, 88.6)	83.1 (82.2, 84.0)	83.5 (82.9, 84.1)
Observed free-roaming dogs	97.0 (95.8, 98.2)	94.9 (92.5, 97.3)	96.7 (95.5, 97.9)	96.6 (95.8, 97.4)
**Confinement of owned dogs (%, 95% CI)**				
No/free-roaming	28.8 (27.7, 30.0)	83.9 (82.7, 85.1)	92.0 (91.4, 92.6)	66.4 (65.6, 67.1)

*^a^Three juveniles were excluded from the analysis due to undetermined sex*.

*^b^Sex ratio by stratum not calculated as a result of limited number of observations*.

Most dogs were reported (owned, 83.5%) or observed (free-roaming, 96.6%) to be adults, ≥1 year old (Table 1). The proportion of adult owned dogs was higher in suburban areas than in urban and rural areas (*p* < 0.001), whereas there were no significant differences in age structure across strata for observed free-roaming dogs (*p* > 0.05).

There was a marked difference in confinement practices reported by dog owners (Table 1). Fewer dogs were allowed to roam in urban (28.8%) than in suburban (83.9%) and rural areas (92.0%, *p* < 0.001). Overall, over 66% of owned dogs were not confined or restricted by their owners.

Four villages, two urban, and two rural experienced dog culling (*n* = 650 dogs killed) within 3 months prior to the DTD survey. All *banjars* within the villages were subject to the culling. There were relatively more juveniles and female dogs in recently culled *banjars* (*p* < 0.05), both urban and rural, areas compared to *banjars* without recent culling (Table 2). No significant demographic differences according to cull status were found in observed free-roaming dogs (*p* > 0.05).

**Table 2:** Demographic characteristics of owned dogs and observed free-roaming dogs in urban and rural ***banjars*** with culling (39 ***banjars***) and without recent culling activities (205 ***banjars***).

	Urban		Rural	
	Non-cull (77)	Cull (23)	Non-cull (128)	Cull (16)
**Sex ratio (male:female)**				
Owned dogs	1.8:1 (*n* = 4,769)	1.5:1 (*n* = 1,836)	3.3:1 (*n* = 6,577)	2.3:1 (*n* = 693)
Observed free-roaming dogs^a^	2.9:1 (*n* = 613)	1.9:1 (*n* = 227)	4.6:1 (*n* = 794)	5.3:1 (*n* = 25)
**Juvenile, <1 year old (%, 95% CI)**				
Owned dogs	17.2 (16.2, 19.3)	21.2 (19.3, 23.1)	16.3 (15.4, 17.2)	22.1 (19.0, 25.2)
Observed free-roaming dogs	3.1 (1.7, 4.5)	2.6 (0.6, 4.7)	3.1 (1.9, 4.4)	8 (0.0, 19.4)
**Confinement of owned dogs (%, 95% CI)**				
No/free-roaming	34.2 (32.8, 35.5)	14.8 (13.1, 16.4)	91.6 (90.9, 92,3)	96.2 (94.8, 97.7)

*^a^Three juveniles were excluded from the analysis due to undetermined sex*.

### Rabies Vaccination Coverage

Rabies vaccination coverage was high in owned dogs (83.6%), but low in observed free-roaming dogs (30.9%, Table 3). The latter was possibly due to loss of collars used to identify the vaccination status of free-roaming dogs, but these may include dogs that were unowned or more difficult to vaccinate. In both dog populations, vaccination coverage was higher in males compared to females (*p* < 0.05) and higher in adult dogs (≥1 year old) than in juveniles (*p* < 0.05). Coverage was also generally better in urban areas compared to suburban and rural areas.

**Table 3:** **Vaccination coverage according to demographic characteristics and environmental setting in dogs from 37 villages in Bali**.

	Urban	Suburban	Rural	Overall
**Total**				
Owned dogs (*n* = 17,376)	88.8 (88.0, 89.6)	83.7 (82.5, 84.9)	78.7 (77.8, 79.6)	83.6 (83.0, 84.2)
Observed free-roaming dogs (*n* = 1,972)	37.5 (34.2, 40.8)	21.4 (16.8, 26.0)	27.8 (24.7, 30.9)	30.9 (28.9, 32,9)
**Male**				
Owned dogs (*n* = 12,228)	90.3 (89.4, 91.2)	86.0 (84.6, 87.3)	80.6 (79.6, 81.7)	85.0 (84.4, 85.6)
Observed free-roaming dogs (*n* = 1,514)	39.5 (35.5, 43.4)	22.9 (17.7, 28.1)	29.9 (26.4, 33.3)	32.4 (30.1, 34.8)
**Female**				
Owned dogs (*n* = 5,148)	86.3 (84.9, 87.7)	77.9 (75.3, 80.5)	72.7 (70.6, 74.8)	80.1 (79.1, 81.2)
Observed free-roaming dogs (*n* = 455)	33.3 (27.5, 39.2)	15.0 (5.9, 24.1)	18.5 (12.2, 24.8)	26.2 (22.1, 30.2)
**Juvenile, <1 year old**				
Owned dogs (*n* = 2,874)	57.5 (54.7, 60.3)	18.0 (14.4, 21.6)	39.8 (37.1, 42.6)	43.9 (42.1, 45.7)
Observed free-roaming dogs (*n* = 68)	20.0 (4.0, 36.0)	0 (0,0)	7.4 (0, 17.5)	10.3 (3.0, 17.6)
**Adult, ≥1 year old**				
Owned dogs (*n* = 14,502)	95.8 (95.3, 96.3)	93.1 (92.2, 94.0)	86.6 (85.7, 87.5)	91.4 (90.9, 91.9)
Observed free-roaming dogs (*n* = 1,904)	38.0 (34.7, 41.3)	22.6 (17.8, 27.4)	28.5 (25.4, 31.7)	31.7 (29.6, 33.8)
**Confinement of owned dogs**				
Confined (*n* = 5,844)	90.6 (89.7, 91.4)	89.9 (87.4, 92.4)	83.0 (80.0, 86.1)	89.8 (89.0, 90.5)
No/free-roaming (*n* = 11,532)	84.4 (82.8, 86.1)	82.6 (81.2, 84.0)	78.4 (77.4, 79.3)	80.4 (79.7, 81.2)
**Recent culling in *banjar***				
Owned dogs (*n* = 2,529)	87.1 (85.6, 88.6)	–	70.7 (67.3, 74.1)	82.6 (81.1, 84.1)
Observed free-roaming dogs (*n* = 252)	51.5 (45.0, 58.1)	–	24.0 (6.9, 41.1)	48.8 (42.6, 55.0)
**No recent culling in *banjar***				
Owned dogs (*n* = 14,84714847)	89.5 (88.6, 90.3)	–	79.6 (78.6, 80.6)	83.7 (83.1, 84.3)
Observed free-roaming dogs (*n* = 1,720)	32.3 (28.6, 36.0)	–	28.0 (24.8, 31.1)	28.3 (26.2, 30.4)

*Coverages are given by % and 95% confidence intervals are shown in brackets*.

In owned dogs, vaccination was reported in 91.4% of adults and 43.9% of juveniles. Meanwhile, in observed free-roaming dogs, the estimated coverage in adults and juveniles was 31.7 and 10.3%, respectively. Low coverage in juveniles was likely due to insufficient targeting of pups and from puppies born subsequent to vaccination campaign. Confined dogs were also more likely to be vaccinated compared to dogs allowed to roam by their owners (*p* < 0.05).

In *banjars* where dogs were subject to recent culling in the last 3 months, vaccination coverage of owned dogs was slightly lower compared to *banjars* without recent culling (*p* < 0.05), but still >70%. Vaccination in observed free-roaming dogs was higher in urban *banjars* subject to recent culling, but not significantly different in the rural *banjars* (*p* < 0.05).

The odds ratios (ORs) of owned dogs not being vaccinated were 19.5, 71.1, and 9.9 in juveniles in urban, suburban, and rural areas, respectively (combined 95% CI: 8.6–96.0), 1.4 in females (95% CI: 1.2–1.5), and 1.4 in dogs in recently culled *banjars* (95% CI: 1.2–1.6) (Table 4). Additionally, the odds of not being vaccinated in free-roaming owned dogs were 3.0 (95% CI: 2.5–3.6) in urban, 3.6 (95% CI: 2.4–5.4) in suburban, and 1.9 (95% CI: 1.4–2.4) in rural areas.

**Table 4:** **Odds ratio (OR) on the risk of not being vaccinated in owned dogs (*n* = 10,352) and observed free-roaming dogs (*n* = 1,972)**.

Variable	OR	
	Owned dogs^a^	Observed free-roaming dogs^b^
**Age**		
Juvenile	–	3.8 (1.7, 8.4)
Juvenile (urban)	19.5 (16.2, 23.4)	–
Juvenile (suburban)	71.1 (52.6, 96.0)	–
Juvenile (rural)	9.9 (8.6, 11.3)	–
**Sex**		
Female	1.4 (1.2, 1.5)	1.6 (1.2, 2.0)
**Recent culling**		
Yes	1.4 (1.2, 1.6)	–
Yes (urban)	–	0.4 (0.3, 0.6)
Yes (rural)	–	1.2 (0.5, 3.0)
**Confinement**		
No/free-roaming (urban)	3.0 (2.5, 3.6)	–
No/free-roaming (suburban)	3.6 (2.4, 5.4)	–
No/free-roaming (rural)	1.9 (1.4, 2.4)	–

*^a^Logistic regression model = −1.9 − 0.8 urban − 0.6 suburban + 2.3 juvenile + 0.3 female + 0.3 culling − 0.6 confinement + 0.7 urban × juvenile + 2.0 suburban × juvenile − 0.5 urban × confinement − 0.7 suburban × confinement*.

*^b^Logistic regression model = 0.8 − 0.3 urban + 0.3 suburban + 1.3 juvenile + 0.4 female + 0.2 culling − 1.0 urban × culling*.

Meanwhile in observed free-roaming dogs, the odds of not being vaccinated were 3.8 times higher in juveniles (95% CI: 1.7–8.4), 1.6 times higher in females (95%: CI: 1.2–2.0), but 0.4 times lower in dogs in recently culled urban *banjars* (95% CI: 0.3–0.6). In rural *banjars*, recent culling was not observed to have a significant effect on rabies vaccination coverage in observed free-roaming dogs (95% CI: 0.5–3.0).

## Discussion

The ongoing rabies epidemic on Bali highlights the major health, economic, and welfare implications of this fatal zoonotic disease (5, 8, 9). Here, we report on surveys of the Bali dog population and resulting recommendations to improve control efforts in Bali and other high-density populations, with large numbers of free-roaming dogs. Overall, we found high but variable numbers of owned dogs, with most allowed to roam freely, except in urban areas where more dogs were reportedly confined. These ownership patterns make estimating vaccine requirements difficult. Loss of vaccination collars means that coverage estimates may not be reliable unless postvaccination surveys were completed rapidly after campaigns and may also lead to a loss of confidence in vaccination. Nonetheless, our estimates of coverage in owned dogs were consistent with those reported after campaigns (5, 9) and were generally high suggesting that rabies will be controlled if these efforts are sustained. We also found that recent culling activities had detrimental effects in owned dog populations and were likely an expensive distraction from vaccination.

Dog ownership in Bali is extremely common. Over 70% of households own dog(s), and Balinese identify dogs as culturally important, with dogs revered in Balinese Hinduism (24). Dogs are reportedly mainly kept to guard the house, with some Balinese believing dogs can alert their owners against evil spirits (24). Our surveys showed that most Bali dogs were allowed to roam (>90% in rural areas, Table 1), which can lead to perceptions of a “stray dog problem” and pose a challenge for achieving and demonstrating high levels of vaccination coverage.

The sex ratio of dogs on Bali was strongly male-biased, although less strong in urban communities, where female dogs appear to be more accepted. This study is consistent with Morters et al. (25) who showed that dogs in Bali are regulated by human demand, with preferences for male dogs. Owner reporting suggests a sex ratio at birth of 1.4:1 (217 puppies from 83 litters); however, even this may be biased with owners reluctant to report dumping of neonates (25). The increasingly biased sex ratio with age suggests higher female mortality across age classes, and focus group discussions indicated a strong preference toward owning male dogs. Such preferences have also been reported from other populations where dogs are mostly free-roaming, including in Madagascar (26), Thailand (27), Mexico (28), and parts of India (29). A greater understanding of mortality determinants should provide insights into demographic turnover, coverage declines between campaigns, and more generally about ownership practices, which could be important to improve vaccine delivery in such populations.

A major limitation of our study was our inability to determine the proportion of the dog population that was truly unowned. DTD surveys captured the population size and characteristics of owned dogs, while PMR transects were suitable for studying free-roaming dogs including unowned dogs. However, it was not possible to determine the ownership status of observed free-roaming dogs. Given the extent to which owned dogs roam freely, it is likely that many of the observed free-roaming dogs were owned dogs. A recent in-depth study on Bali illustrated that contrary to appearances, almost all dogs are owned (14). The higher proportion of observed adult dogs’ free-roaming in our study in comparison to owned dogs is likely due to dog behavior, as our own unpublished data on dog movement show that juveniles were more likely to remain near their homestead in comparison to adult dogs.

Another limitation to our study was potential bias from misclassification of vaccination status. For owned dogs, vaccination status was determined based on owner report. Despite vaccination cards given to owners during mass dog vaccination campaigns, many were misplaced or lost and reporting relied heavily on memory. Vaccination coverage reported in owned dogs was very high (80%), with small but significant differences according to the setting (urban, suburban, and rural), sex, and ownership practices. The lower perceived value of female dogs, evident from the male-biased sex ratio, might have influenced dog owners’ effort to vaccinate their animals (30). Vaccination coverage was also significantly lower in juvenile dogs (18–57%), particularly those in suburban and rural settings, and below the threshold coverage (70%) required to control rabies (6). The risk of juvenile dogs not being vaccinated was very high (OR: 10–70). Similar situations were found in Mexico and Bolivia where juveniles have a higher risk of not being vaccinated (28, 31). Low vaccination coverage in juveniles was likely due to insufficient targeting of pups. It is a common perception that puppies cannot be vaccinated because they are too young, but even extremely young dogs in rabies endemic regions have been shown to respond very well to vaccination (32). Although some juveniles may not have been born at the time of the campaign, many could have been vaccinated leading to improved coverage (>15% of the dog population were <1 year).

In contrast, the vaccination status in observed free-roaming dogs was determined by the presence/absence of vaccination collar. The estimated coverage was very low (20–40%), particularly in juveniles. Collars used during the first vaccination campaign in Bali were reportedly lost rapidly. Although the quality of collars used subsequently improved, it is not known how long collars last. Collar-based postvaccination surveys carried out some time after vaccinations were therefore likely to underestimate coverage. This is another limitation to our survey as transects were not always conducted immediately after vaccination in a *banjar*. Transects carried out immediately after campaigns enable more reliable estimates of coverage (5), but long-lasting, cheap, and quick ways of marking vaccinated dogs are still needed to instill confidence in communities that dogs remain protected.

Culling is commonly used in many developing countries in response to rabies, despite being ineffective (33). Our data show that in *banjars* subject to recent culling, the proportion of owned juvenile dogs was significantly higher (Table 2); however, overall vaccination coverage of owned dogs was significantly lower (Table 3), than in *banjars* not subject to culling. There could be several reasons for this: owners might replace culled animals (typically with unvaccinated juvenile animals), while older (free-roaming) animals might be easier to cull than juveniles. In urban areas, confinement of owned dogs in culled villages was also higher (by 15%), which may be in response to the threat of culling. The percentage of observed free-roaming dogs with vaccination collars was also higher by 20% in urban *banjars* subject to culling, whereas no difference was observed in rural *banjars* (Table 3). However, as discussed already, vaccination collars may not be a reliable indicator of coverage. Moreover, the PMR surveys provided data on a potentially smaller segment of the dog population than DTD surveys. These data do not provide any support for culling and instead indicate potentially detrimental effects, including increased susceptibility in some communities.

Overall, we found high vaccination coverage among dogs on Bali, which is promising for prospects of eliminating rabies from Bali. We did find significant risk factors for non-vaccination, which likely contribute to ongoing rabies persistence and should be prioritized in future control efforts. First, coverage was lowest in rural dog populations, where most dogs were unconfined, and where typically more rabies circulates (34). Juvenile dogs were also least likely to be vaccinated. Targeted efforts to vaccinate free-roaming dogs in rural populations and especially puppies are therefore recommended. Post-campaign efforts to vaccinate any new puppies should also be encouraged to try to reduce susceptibility gaps. Moreover, we did not find any evidence on the positive impacts of culling on vaccination coverage, consistent with other research (33). Therefore, we recommend that control should focus on vaccination, which has been proven effective (35). Previous work on Bali demonstrated that virus transmission can be sustained in local communities missed by vaccination campaigns (5). However, by targeting these risk groups (free-roaming dogs, particularly puppies in rural and suburban areas) and ensuring all populations are vaccinated, sustained vaccination effort should lead to the elimination of rabies from Bali.

## Ethics Statement

This study was carried out with permission and endorsement from Bali Provincial Livestock and Animal Health Office and acknowledgment from the Directorate of Animal Health, Directorate General of Livestock and Animal Health Services, Ministry of Agriculture, Republic of Indonesia. Ethical clearance from the Ministry of Health was not required because people were interviewed only in their capacity as dog owners.

## Author Contributions

RA and KH were the main authors of this paper. RA, KH, AJ, MW, SS, CB, AP, IW, and AE were essential in the planning, designing, and implementation of the research. IM, IK, and IS were critical members of the local authorities who supported field implementation and provided input to the research. PD and MS guided data analysis and interpretation. JG and FU are from ILRI through which this research was funded.

## Conflict of Interest Statement

The authors declare that the research was conducted in the absence of any commercial or financial relationships that could be construed as a potential conflict of interest.
