# Corrigendum: Determinants of Vaccination Coverage and Consequences for Rabies Control in Bali, Indonesia

**DOI:** 10.3389/fvets.2017.00020

**Published:** 2017-02-22

**Authors:** Riana A. Arief, Katie Hampson, Andri Jatikusumah, Maria D. W. Widyastuti, Sunandar, Chaerul Basri, Anak A. G. Putra, Iwan Willyanto, Agnes T. S. Estoepangestie, I. W. Mardiana, I. K. G. N. Kesuma, I. P. Sumantra, Paul F. Doherty, M. D. Salman, Jeff Gilbert, Fred Unger

**Affiliations:** ^1^Center for Indonesian Veterinary Analytical Studies, Bogor, Indonesia; ^2^Boyd Orr Centre for Population and Ecosystem Health, Institute of Biodiversity, Animal Health and Comparative Medicine, University of Glasgow, Glasgow, UK; ^3^Department of Animal Disease and Veterinary Public Health, Faculty of Veterinary Medicine, Bogor Agricultural University, Bogor, Indonesia; ^4^Denpasar Disease Investigation Center, Denpasar, Indonesia; ^5^InI Veterinary Service, Surabaya, Indonesia; ^6^Department of Veterinary Public Health, Faculty of Veterinary Medicine, Airlangga University, Surabaya, Indonesia; ^7^Bali Provincial Livestock and Animal Health Office, Denpasar, Indonesia; ^8^Department of Fish, Wildlife and Conservation Biology, Warner College of Natural Resources, Colorado State University, Fort Collins, CO, USA; ^9^Animal Population Health Institute, College of Veterinary Medicine and Biomedical Sciences, Colorado State University, Fort Collins, CO, USA; ^10^International Livestock Research Institute, Hanoi, Vietnam

**Keywords:** rabies, dogs, vaccination, questionnaire survey, mark–recapture survey, Bali, Indonesia

**Incorrect Funding Number**

There is an error in the Funding statement. The correct number for **Wellcome Trust** is **095787/Z/11/Z to KH**. The authors apologize for this error and state that this does not change the scientific conclusions of the article in any way.

## Conflict of Interest Statement

The authors declare that the research was conducted in the absence of any commercial or financial relationships that could be construed as a potential conflict of interest.

